# Local delivery of fluorescent dye for fiber-optics confocal microscopy of the living heart

**DOI:** 10.3389/fphys.2014.00367

**Published:** 2014-09-25

**Authors:** Chao Huang, Aditya K. Kaza, Robert W. Hitchcock, Frank B. Sachse

**Affiliations:** Department of Bioengineering, University of UtahSalt Lake City, UT, USA; Department of Cardiac Surgery, Boston Children's Hospital and Harvard Medical SchoolBoston, MA, USA; Nora Eccles Harrison Cardiovascular Research and Training Institute, University of UtahSalt Lake City, UT, USA

**Keywords:** fiber-optics confocal microscopy, cardiac microscopy, fluorescent dyes, dye carrier, cardiac surgery

## Abstract

Fiber-optics confocal microscopy (FCM) is an emerging imaging technology with various applications in basic research and clinical diagnosis. FCM allows for real-time *in situ* microscopy of tissue at sub-cellular scale. Recently FCM has been investigated for cardiac imaging, in particular, for discrimination of cardiac tissue during pediatric open-heart surgery. FCM relies on fluorescent dyes. The current clinical approach of dye delivery is based on systemic injection, which is associated with high dye consumption, and adverse clinical events. In this study, we investigated approaches for local dye delivery during FCM imaging based on dye carriers attached to the imaging probe. Using three-dimensional confocal microscopy, automated bench tests, and FCM imaging we quantitatively characterized dye release of carriers composed of open-pore foam only and foam loaded with agarose hydrogel. In addition, we compared local dye delivery with a model of systemic dye delivery in the isolated perfused rodent heart. We measured the signal-to-noise ratio (SNR) of images acquired in various regions of the heart. Our evaluations showed that foam-agarose dye carriers exhibited a prolonged dye release vs. foam-only carriers. Foam-agarose dye carriers allowed reliable imaging of 5–9 lines, which is comparable to 4–8 min of continuous dye release. Our study in the living heart revealed that the SNR of FCM images using local and systemic dye delivery is not different. However, we observed differences in the imaged tissue microstructure with the two approaches. Structural features characteristic of microvasculature were solely observed for systemic dye delivery. Our findings suggest that local dye delivery approach for FCM imaging constitutes an important alternative to systemic dye delivery. We suggest that the approach for local dye delivery will facilitate clinical translation of FCM, for instance, for FCM imaging during pediatric heart surgery.

## Introduction

Fiber-optics confocal microscopy (FCM) is based on confocal microscopy, which was invented and patented in the 1950s by Minsky (1961). Similarly to conventional confocal microscopy, FCM allows for high-resolution optical imaging at various depths within a specimen. With FCM, it is possible to visualize cellular and sub-cellular features within approximately 100 μm from a specimen's surface. In contrast to conventional confocal microscopic systems FCM systems comprise a coherent fiber-optic bundle with a length of up to several meters. In many implementations, an imaging microprobe including a lens is located at the tip of the fiber-optic bundle. The detachment of the imaging microprobe from other components of the imaging system allows *in situ* imaging of organs and tissues within a body, which cannot be performed with conventional microscopy. In the last years, the unique capability of real-time, *in situ* microscopic imaging has led to development of FCM-based approaches for clinical diagnosis in gastrointestinology (Inoue et al., 2005; Kiesslich and Neurath, 2005; Goetz et al., 2006; Anandasabapathy, 2008), pulmonology (Thiberville et al., 2009; Salaun et al., 2013), and urology (Wu et al., 2011).

Recently, we introduced FCM for imaging of the living heart and developed approaches for tissue discrimination during cardiac interventions (Lasher et al., 2009; Huang et al., 2013). In particular, we proposed application of FCM during pediatric open-heart surgery for repair of congenital heart defects. Injury to specialized tissue of the conduction system is a major risk in surgical repair of congenital heart defects. In order to avoid damaging the conduction system, surgeons rely on an empirical approach and guidelines developed from gross anatomical studies to approximate the disposition of the conduction system. However, the distribution of the conduction system can vary individually and with the complexity of the heart defect (Anderson et al., 1983; Andersen et al., 2006). Our previous studies indicate feasibility of intraoperative discrimination of cardiac tissue using FCM, which will reduce the risk of injury to the specialized tissue of the conduction system (Huang et al., 2013). In these studies we investigated sinoatrial and atrioventricular node tissue as well as atrial working myocardium. Tissue discrimination was performed based on microstructural features characteristic of these tissue types and immunohistochemical markers.

A major challenge in applying FCM for intraoperative microscopic imaging is the reliance of the technology on fluorescent labeling. FCM illuminates fluorescent molecules within the imaged specimen using a light source, commonly at a wavelength 488 or 633 nm. The light is absorbed by fluorescent molecules, which in turn emit light at a longer wavelength. This emitted light is collected through the fiber-optic bundle and visualized by the FCM system. Generally, intrinsic fluorescence in biological specimens is low and labeling of specimens with fluorescent dyes is required for imaging. The common approach for dye delivery in clinical applications of FCM is systemic injection of fluorescent dye at high concentrations. In addition to high dye consumption, there have been also some concerns of patient safety. For example, the incidence of adverse reactions following intravenous administration of fluorescein is 1.1–4.8% (Kwiterovich et al., 1991; Kwan et al., 2006) for retinal fluorescein angiography and 1.4% for gastrointestinal endoscopic imaging (Wallace et al., 2010). In these studies, adverse reactions including dizziness, nausea, vomiting, and transient hypotension have been reported. However, there were no serious adverse reactions or deaths. We suggest that a more localized dye delivery approach may serve as an alternative (Lasher et al., 2009) and be well suited for intraoperative imaging applications such as in pediatric open-heart surgery or various other surgical disciplines (Sachse et al., 2011, 2013).

In this study, we implemented and evaluated approaches for delivery of fluorescent dye during FCM imaging in the living heart. In particular, we compared approaches of local dye delivery with a model of systemic dye delivery. For this purpose, we investigated dye release characteristics and quality of images produced using several local dye delivery methods and fluorescent dyes. These local dye delivery methods were based on dye carriers assembled from medical grade foam and agarose hydrogel. The dye carriers were attached to the tip of the FCM imaging microprobe in such a manner that placement of the assembly onto the surface of a specimen resulted in continuous release of fluorescent dye from the carrier to the specimen throughout the image acquisition. Using three-dimensional confocal microscopy, automated bench tests, and FCM, we quantitatively identified carriers with release characteristics suitable for intraoperative imaging. Local dye delivery using dye carriers was subsequently compared with a model of systemic dye delivery in isolated perfused heart of a small mammal based on FCM imaging and different fluorescent dyes. The isolated hearts were arrested to reproduce cardioplegia in open-heart surgery. Two-dimensional image sequences were acquired with FCM from the ventricular and atrial subepicardial myocardium as well as the sinoatrial node region. We assessed image quality based on analysis of signal-to-noise ratio from FCM images acquired in these regions of the heart using both dye delivery approaches.

## Materials and methods

### Dye carrier material and fabrication

Several types of dye carriers were fabricated from polyester polyurethane foam with a density of 27.7–33.0 kg/m^3^ and 31.5–39.4 pores/cm (FLTZ90D; Foamex Innovations Inc., Ft. Wayne, IN, US). The foam material was reticulated so that pores within the material are open and interconnected. The fabrication of the dye carrier involved boring the inner channel and main body of foam cylinders using biopsy punches with 2 and 6 mm diameter (BP99; HealthLink, Jacksonville, FL, US), respectively, from stock 15 by 15 by 1.2 cm foam sheets. For some dye carriers the foam cylinders were loaded with agarose gel. The gel was made by mixing agarose powder (GeneMate LE Agarose; BioExpress, Kaysville, UT) in distilled water to concentrations of 1, 3, or 5%. The agarose powder in solution was dissolved for 15 min at 75–80°C with continuous magnetic stirring. Immediately following dissolution, the agarose solution was filled into Eppendorff tubes (1.5 ml). Foam cylinders were immersed in the gel and compressed with forceps for 1 min to remove air bubbles. The gel was cured at room temperature for 15 min followed by overnight incubation at 4°C. The following day, excess agarose gel within the inner channel and encapsulating the foam-agarose cylinders were removed. Foam-only and foam-agarose carriers containing 1, 3, and 5% agarose gel were cut from the cylinders with a razor blade to a height of 4 mm.

### Dye carrier and dye preparation

Foam-only carriers were repetitively compressed for 1 min in distilled water containing 10 kDa dextran conjugated Alexa Fluor 488 (Invitrogen, Carlsbad, CA, US) at a 125 μg/ml concentration followed by immersion in the dye solution for at least 15 min prior to imaging. Foam-agarose carriers were similarly immersed for 15 min, but without compression, in distilled water containing dextran or fluorescein sodium [Fluorescite® (fluorescein injection, USP) 10%; Alcon, Fort Worth, TX, US; 1:1000]. For living arrested heart preparations, foam-agarose carriers were immersed for 15 min in oxygenated, high-K/low-Ca Tyrode's solution (in mmol/L: 92 NaCl, 11 dextrose, 13.2 KCl, 5 MgCl_2_, 24 HEPES, 20 taurine, 5 creatine, 5 C_3_H_3_NaO_3_, 1 NaH_2_PO4, 0.25 CaCl_2_; pH 7.4; ≈10°C) containing fluorescein sodium (1:1000). For experiments involving dye perfusion in the living arrested heart, a perfusate of oxygenated, high-K/low-Ca Tyrode's solution containing fluorescein sodium (1:50000) was prepared.

### FCM imaging

Two-dimensional image sequences of synthetic tissue and preparations of living cardiac tissue were acquired with a FCM system (FCM 1000; Leica Microsystems GmbH, Wetzlar, Germany) equipped with a custom fiber-optics microprobe (UltraMiniOWD30; Mauna Kea Technologies, Paris, France). The resolution was 1.9–2.4 μm in x- and y-direction and approximately 10 μm in z-direction. The field of view (xy) was 186 by 130 μm at a depth (z) of approximately 26 μm. The frame rate was 12 Hz.

### Evaluation of dye release

**Figure 1:**
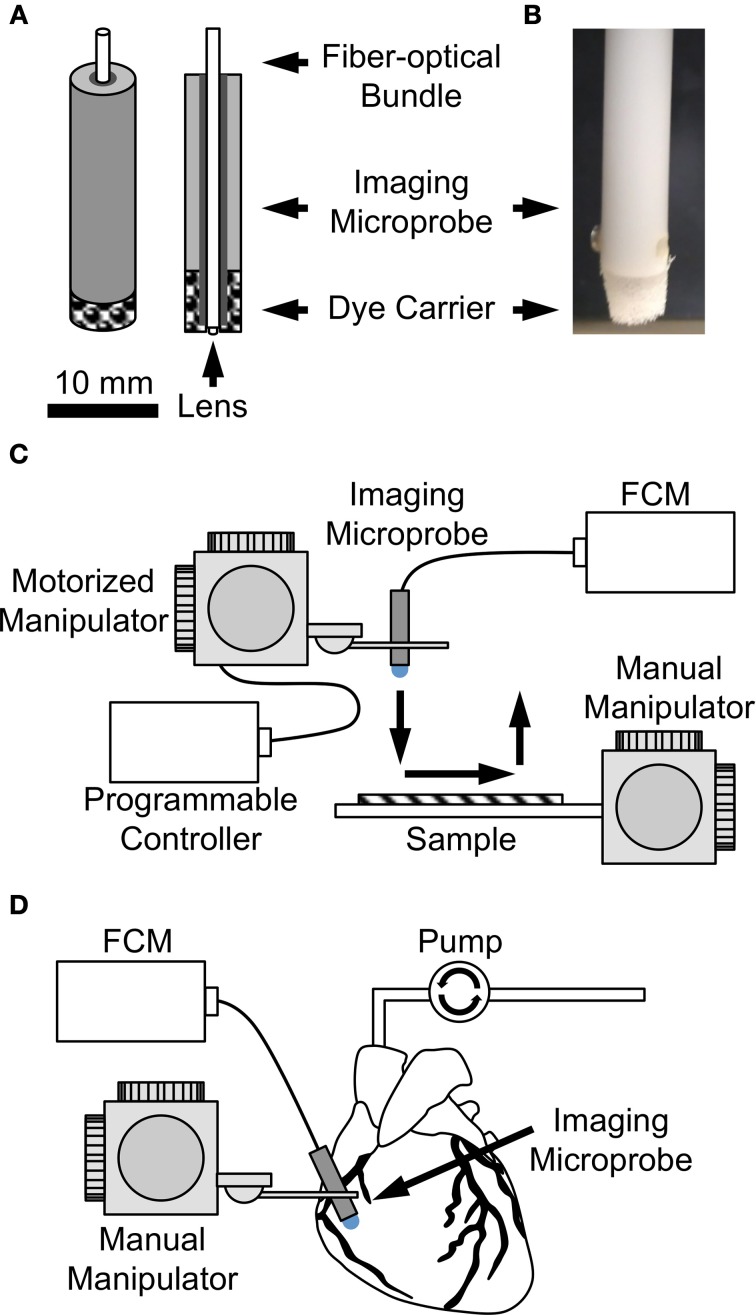
**(A)** Sketch and **(B)** photo of dye carrier attached to imaging microprobe. Dye carriers were assembled on the fiber-optics microprobe so that the probe tip was flush with the carrier. Imaging setup for evaluation of dye delivery methods **(C)** on the bench and **(D)** in the living arrested heart.

In a first set of experiments, we performed weight measurements on foam-only carriers (*n* = 3) and foam-agarose carriers with 1, 3, and 5% agarose concentrations (*n* = 3 for each concentration). Carriers of these types were weighed (BP211D, Sartorius, Elk Grove, IL, USA) before and after loading with dextran-Alexa 488. In another set of experiments, the same types of carriers were loaded with dextran-Alexa 488 or fluorescein sodium solution and subjected to cyclic line tests to evaluate dye release. In short, loaded dye carriers were assembled on the fiber-optics microprobe so that the probe tip was flush with the carrier (Figures 1A,B). The assembled probe with dye carrier was then attached to a motorized micro-manipulator (MP285; Sutter Instrument, Novato, CA, US) allowing for programmed movements in three-dimensional space at high resolution. A testbed attached to a manual manipulator (World Precision Instruments, Inc., Sarasota, FL, US) was placed underneath the microprobe. Samples of moisturized synthetic tissue analog (VWR, West Chester, PA, US) composed of 100% virgin wood fiber were affixed to the testbed and brought into focus underneath the imaging probe at the correct focal depth using the manual manipulator. Immediately following, programmed movement of probe across the surface of the sample was initiated. Specifically, the micro-manipulator was programmed to contact the microprobe with attached dye carrier to the surface of the sample, move the probe 5 mm across the surface along a linear path at a speed of 100 μm/s, remove the probe from the sample, and return the probe to the original starting position. A schematic of the cyclic line test is shown in Figure 1C. Loaded dye carriers were subjected to a continuous cycle of line tests with a fresh synthetic tissue sample being presented after each line. 2D image sequences of each line were recorded until image quality became severely reduced.

#### Microscopic imaging of dye carriers

In some experiments, carriers loaded with fluorescent dye and subjected to a cyclic line test were imaged using a laser scanning confocal microscope (Zeiss LSM 5 Duo; Zeiss, Jena, Germany) equipped with a 2.5× lens with a numerical aperture of 0.12. Fluorophores within the carriers were excited with a 488 nm wavelength laser and 3D image stacks were acquired at a spatial resolution of 10 × 10 × 20 μm in x-, y-, and z-direction, field of view (xy) of 5.1 by 5.1 mm, and depth (z) up to 500 μm. Cross-sections through the surface of different carriers were compared.

#### Quantitative analysis of dye release in bench tests

The dye release characteristic of different dye carriers was analyzed using the 2D image sequences of synthetic tissues. For this purpose, the image sequences were converted using MATLAB (MathWorks, Natick, MA, US). The average and standard deviation of the intensity for each image within a line was determined and averaged across the whole line. The average intensities for each line in a cyclic line test are denoted as *I_lines_* = {*I*_1_, *I*_2_, …, *I_n_*} with the last line of a given cyclic line test *n*. *I_lines_* was then smoothed using a central moving average of 1 line. In addition, the sample mode *I_mode_* and standard deviation *I_std_* of the background were calculated for each cyclic line test based on an image acquired in air at the end of each test. *I_mode_* was determined from the maximum in the image histogram. The threshold intensity, *I_thresh_*, for each cyclic line test was then calculated:

$$I_{thresh}=I_{mode}+\gamma I_{std}.$$

We chose γ = 10 based on visual inspection of a subset of image sequences. We found that images with γ greater than 10 exhibited clearly identifiable microstructures that were not identifiable in images with γ below this value. *I_thresh_* was used to quantitatively assess image quality. The number of lines *L_thresh_* that were drawn before *I_lines_* of a cyclic line test reached *I_thresh_* was used to evaluate dye release of various dye carriers and solutions. Table 1 shows sample sizes for the dye carriers and solutions evaluated.

**Table 1:** **Number of Cyclic Line Tests Performed for Evaluation of Dye Release**.

**Fluorescent dye**	**Carrier**		
	**1%**	**3%**	**5%**
Dextran-Alexa 488	11	8	9
Fluorescein sodium	10	8	9

### Evaluation of dye delivery approaches in the living heart

All procedures were approved by the University of Utah Institutional Animal Care and Use Committee and followed the guidelines of the National Institutes of Health Guide for the Care and Use of Laboratory Animals. Sprague-Dawley rats of approximately 300 g body weight were anesthetized with pentobarbital (40 mg/kg) and anticoagulated with heparin (500 IU/kg). Following anesthesia, the hearts were rapidly excised and continuously Langendorff-perfused (Langendorff, 1895) with oxygenated, high-K/low-Ca Tyrode's solution at a flow rate of 10–15 mL/min.

Two methods of dye delivery were subsequently evaluated. The first method was based on delivery of dye via perfusion of the Langendorff heart preparation as a model of systemic dye delivery. In short, perfusion was switched after 5 min to Tyrode's solution containing fluorescein sodium. Immediately following, 2D image sequences of ventricular and atrial subepicardial myocardium and of the sinoatrial node region were acquired. The second method was based on the dye carriers described above. The heart was continuously Langendorff-perfused with Tyrode's solution. Image sequences of the above-mentioned regions were acquired using an imaging microprobe with attached foam-agarose carrier of 1% agarose concentration loaded with fluorescein sodium (Figure 1D).

#### Processing and visualization of images

2D images sequences were converted using MATLAB. Images auto-adjusted for brightness and contrast using the imadjust function in MATLAB.

#### Quantitative analysis of dye delivery approaches

The signal-to-noise ratio (SNR) was calculated from image sequences acquired of various tissue regions using both dye delivery approaches: dye carrier and perfusion. In general, SNR is defined as μ/σ where μ is the mean of the signal and σ is an estimate of the standard deviation of the noise. In this study, a region of high and low signal was selected from each image. Each region comprised approximately 1000 pixels and corresponded to an area of signal or background. The mean intensity within these areas of signal and background was calculated and denoted as *I_sig_* and *I_bg_*, respectively. In addition, the standard deviation within the background region *I_std_* was determined. The SNR for each image was calculated as:

$$SNR=\left(I_{sig}-I_{bg}\right)/I_{std}$$

The SNR was used to evaluate dye delivery based on dye carrier and perfusion approaches in various tissue regions.

### Imaging of microvasculature in the living heart

Two-dimensional image sequences in an arrested Langendorff-perfused rat heart labeled with high molecular weight dextran conjugate in perfusate were acquired using FCM. In short, a perfusate of oxygenated, high-K/low-Ca Tyrode's solution containing 2 MDa dextran conjugated to fluorescein (Invitrogen) at a concentration of 40 μg/mL was prepared. For full dissolution of the dextran-conjugate, the perfusate was vortexed for 5 min followed by sonication (Model 150V/T Ultrasonic Homogenizer; Biologics Inc., Cary, NC, US) at 50% power with a pulse width and pulse duration of 1 s and 2 s respectively for 5 min. Immediately following, the perfusate was centrifuged (Allegra X-22; Beckman Coulter, Indianapolis, IN, US) for 5 min at 6000 *g* and then passed through a 0.22 μm syringe filter (Millex-GP; Millipore, Billerica, MA, US). Images from the right ventricular and atrial regions in the living heart were acquired using the perfusion method of dye delivery as previously described.

### Statistical analysis

Statistical data are presented as mean ± s.e.m. Statistical significance was assessed by One-Way analysis of variance (ANOVA) followed by *post-hoc* Tukey–Kramer considering a significance level of 0.01.

## Results

### Evaluation of dye release in bench tests

In a first set of experiments, we explored structural and functional properties of foam-only and foam-agarose carriers using weight measurements laser scanning confocal microscopy, and cyclic line tests. Our aim was to identify dye carriers, which maintain a high volume of dye retention and prolonged rate of dye release during cyclic line tests.

**Figure 2:**
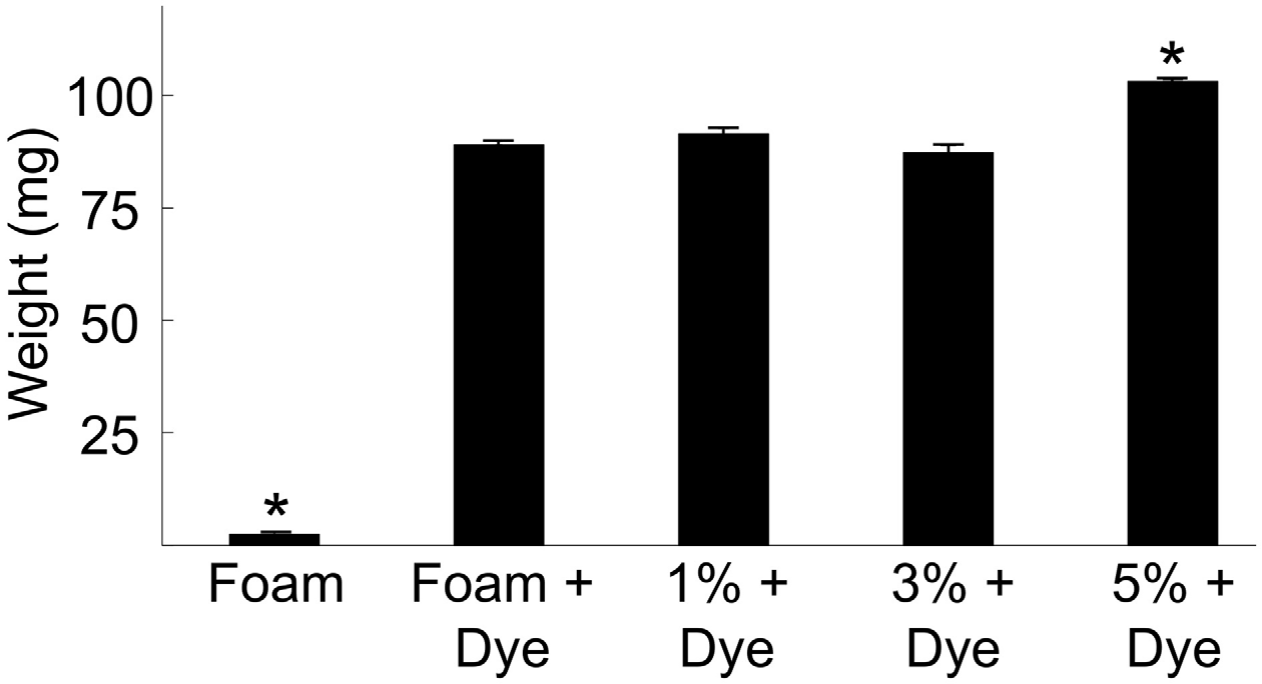
**Weights of various foam-only and foam-agarose carriers before and after loading of dextran-Alexa 488**. Foam-only carriers exhibited a 34-fold increase in weight after dextran-Alexa 488 was loaded. We observed only small differences in the weight of various foam-only and foam-agarose carriers after loading of dextran-Alexa 488. (^*^P < 0.01, significant difference vs. all other groups, One-Way ANOVA with *post-hoc* Tukey–Kramer).

We weighed foam-only and foam-agarose carriers of 1, 3, and 5% agarose concentration before and after immersion in dextran-Alexa 488. Statistical analysis of the measured weights is presented in Figure 2. The weight of the foam-only carrier increased from 2.52 ± 0.03 to 89.00 ± 0.79 mg after loading of dextran, an approximately 34-fold increase in weight. In general, the variation in the weight of the foam-only and foam-agarose carriers after loading of dextran-Alexa 488 was small with weights ranging from approximately 87–103 mg.

**Figure 3:**
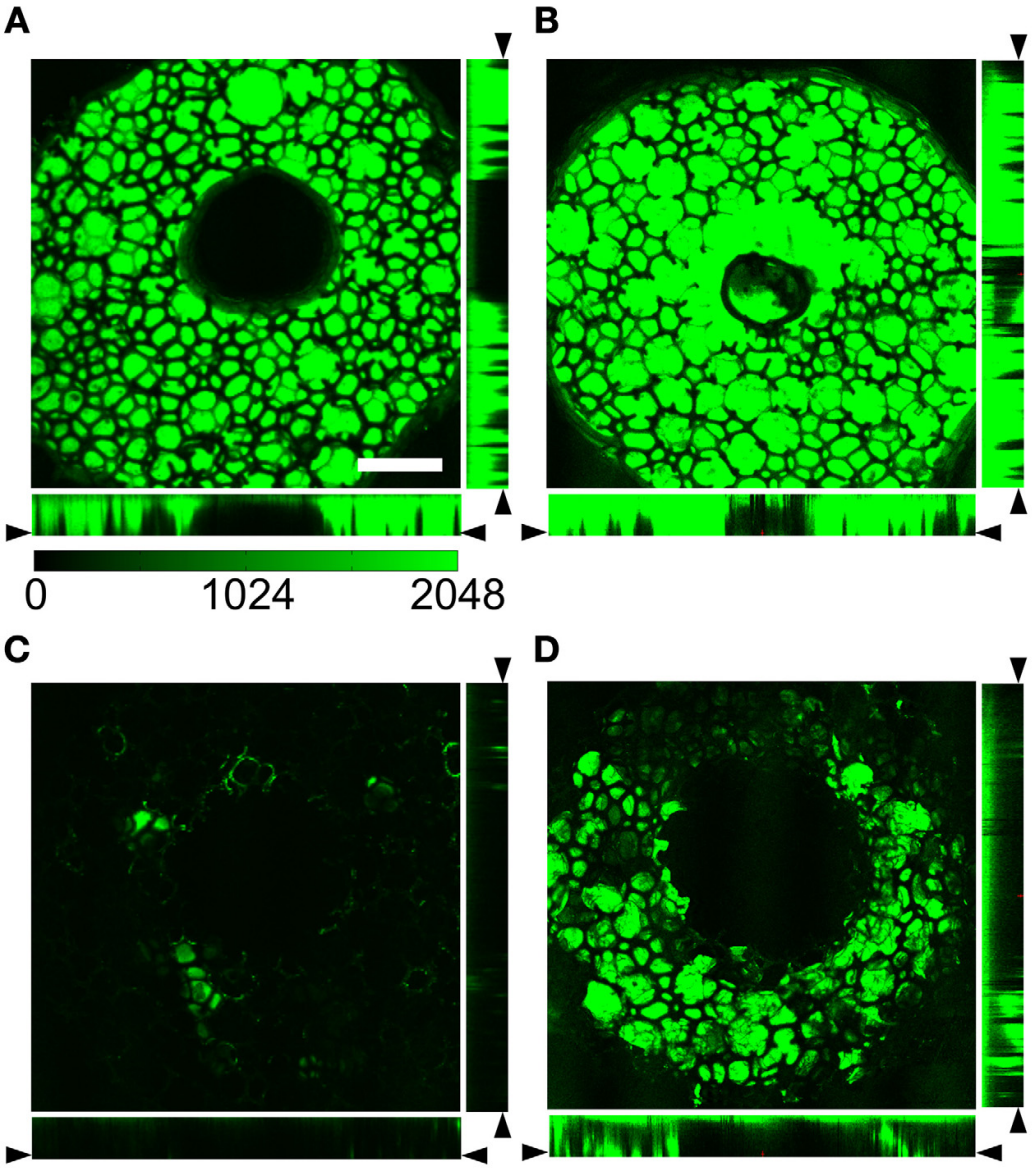
**Cross-sections through image stacks acquired with laser scanning confocal microscopy of (A,B) foam-only and (C,D) 1% foam-agarose carrier loaded with dextran-Alexa 488**. Foam-only and 1% foam-agarose carrier **(A,C)** before and after **(B)** one and **(D)** seven lines, respectively. Dye is almost completely released after a single line in the foam-only carrier. Scale: 1 mm. (Images modified from Sachse et al., 2013).

We then acquired 3D image stacks of the distal end of foam-only and foam-agarose carriers with 1% agarose concentration loaded with dextran-Alexa 488. Cross-sections through those stacks are shown in Figures 3A,B, respectively. The cross-sections illustrate the porous and reticulated microstructure of the foam material, which has highly variable pore sizes in the sub-millimeter scale. Dye distribution was homogeneous in the interior of the foam. The agarose gel did not appear to affect the structural properties of the carriers as both the foam-only and 1% foam-agarose carrier exhibited similar microstructural characteristics and fluorescence intensities.

We further subjected the foam-only and 1% foam-agarose carrier to cyclic line tests to characterize dye release. The fluorescent signal was greatly reduced in the foam-only carrier after only one line (Figures 3C vs. 3A). Only small regions with dye remained, strongly suggesting that most of the dye was transferred to the synthetic tissue. The fluorescent intensity within the 1% foam-agarose carrier, however, was partially maintained even after seven lines of a cyclic line test (Figures 3D vs. 3B). Clearly, the foam-only and 1% foam-agarose carrier exhibited different release properties. The dye release was dramatically slowed for the foam-agarose carrier as compared to the foam-only carrier after cyclic line test. Foam-only carriers exhibited a severe intensity decrease after the first line of a cyclic line test, which makes them unsuitable for applications such as cardiac surgery requiring several minutes of dye release. Based on this finding we excluded foam-only carriers from further assessment.

**Figure 4:**
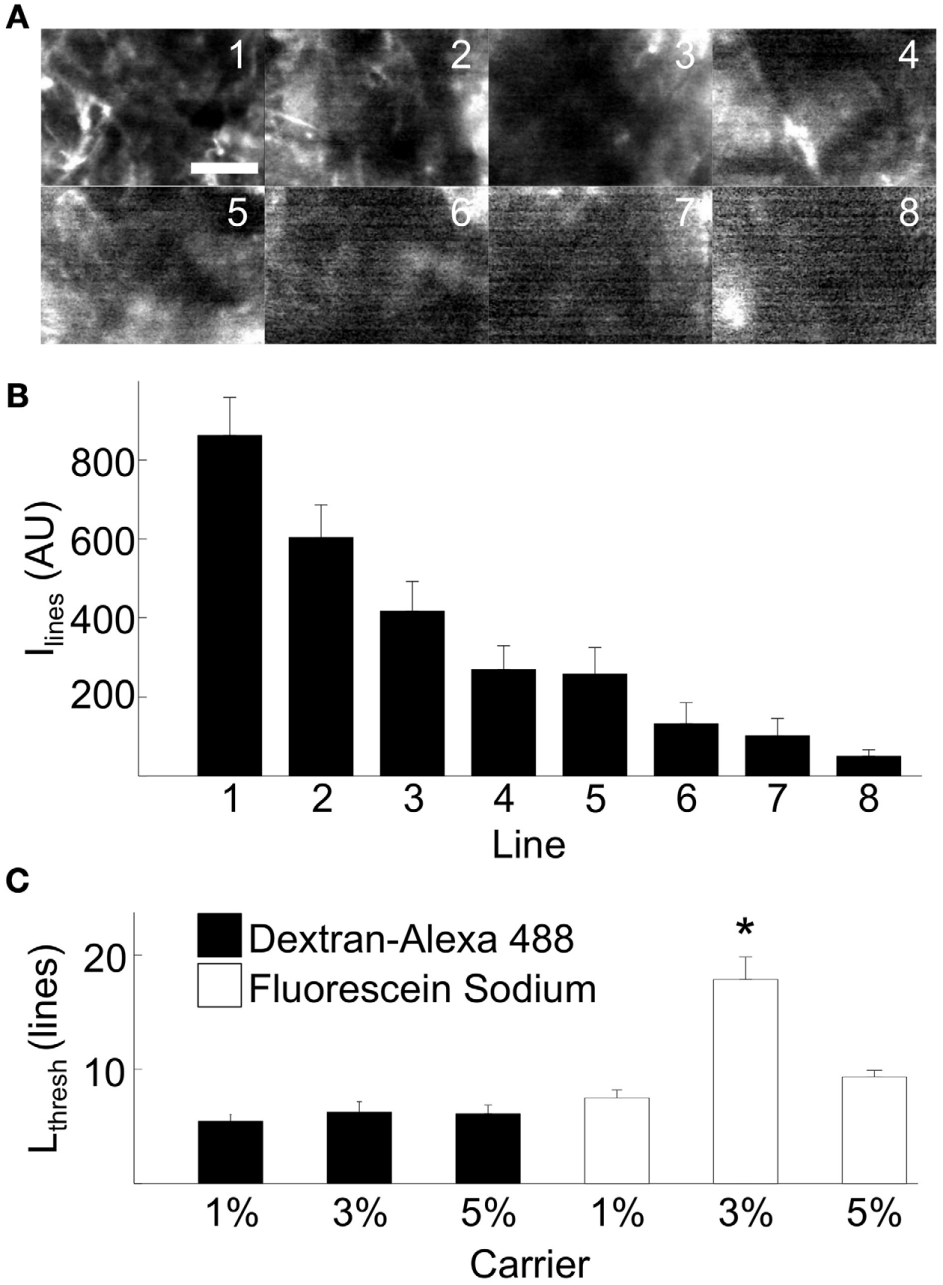
**Experimental evaluation of dye release from carrier on the bench**. **(A)** Example FCM images from cyclic line test on a 1% foam-agarose carrier loaded with dextran-Alexa 488 showing decreasing SNR. Scale: 50 μm. **(B)** Mean and standard deviation of intensities for lines 1–8 in **(A)**. An *L_thresh_* = 5 was determined from *I*_thresh_ = 207.3 AU for the cyclic line test. **(C)** Statistical analysis of dye release of various foam-agarose carriers and fluorescent dyes. The 3% carrier loaded with fluorescein sodium was observed to produce 2–3 times higher *L_thresh_* than any other combination of carrier or dye. (^*^P < 0.01, significant difference vs. all other groups, One-Way ANOVA with *post-hoc* Tukey–Kramer).

We further characterized the functional properties of foam-agarose carriers and fluorescent dyes with the cyclic line tests. We acquired FCM image sequences of synthetic tissue using foam-agarose carriers with 1, 3, and 5% agarose concentrations loaded with either dextran-Alexa 488 or fluorescein sodium (Table 1). Figure 4A presents FCM images from an example cyclic line test of a 1% agarose carrier loaded with dextran-Alexa 488. Figure 4B shows the average and standard deviation of intensities for each line of the example test in Figure 4A. In this example, the average intensity of each line gradually decreased from 862.4 ± 95.9 arbitrary units (AU) to 49.9 ± 16.3 AU from line 1 to line 8, respectively. The decrease of average intensity was approximately exponential. An *L_thresh_* of 5 lines was determined from an *I_thresh_* of 207.3 AU. Features of the imaged sample were still discernable at line 5 with a measured average intensity and standard deviation of 257.3 ± 67.3 AU (Figure 4A).

Statistical analyses of *L_thresh_* from cyclic line tests are presented in Figure 4C. In general, the foam-agarose carriers exhibited similar dye release properties allowing for 5–9 lines before a decrease of signal intensity beyond *I_thresh_*. An exception was the 3% carrier loaded with fluorescein sodium, which allowed for 17.9 ± 2.0 lines before signal decrease. In all but this case, differences in *L_thresh_* were not significant between any other combination of foam-agarose carriers and fluorescent dye.

### Evaluation of dye delivery approaches in the living heart

**Figure 5:**
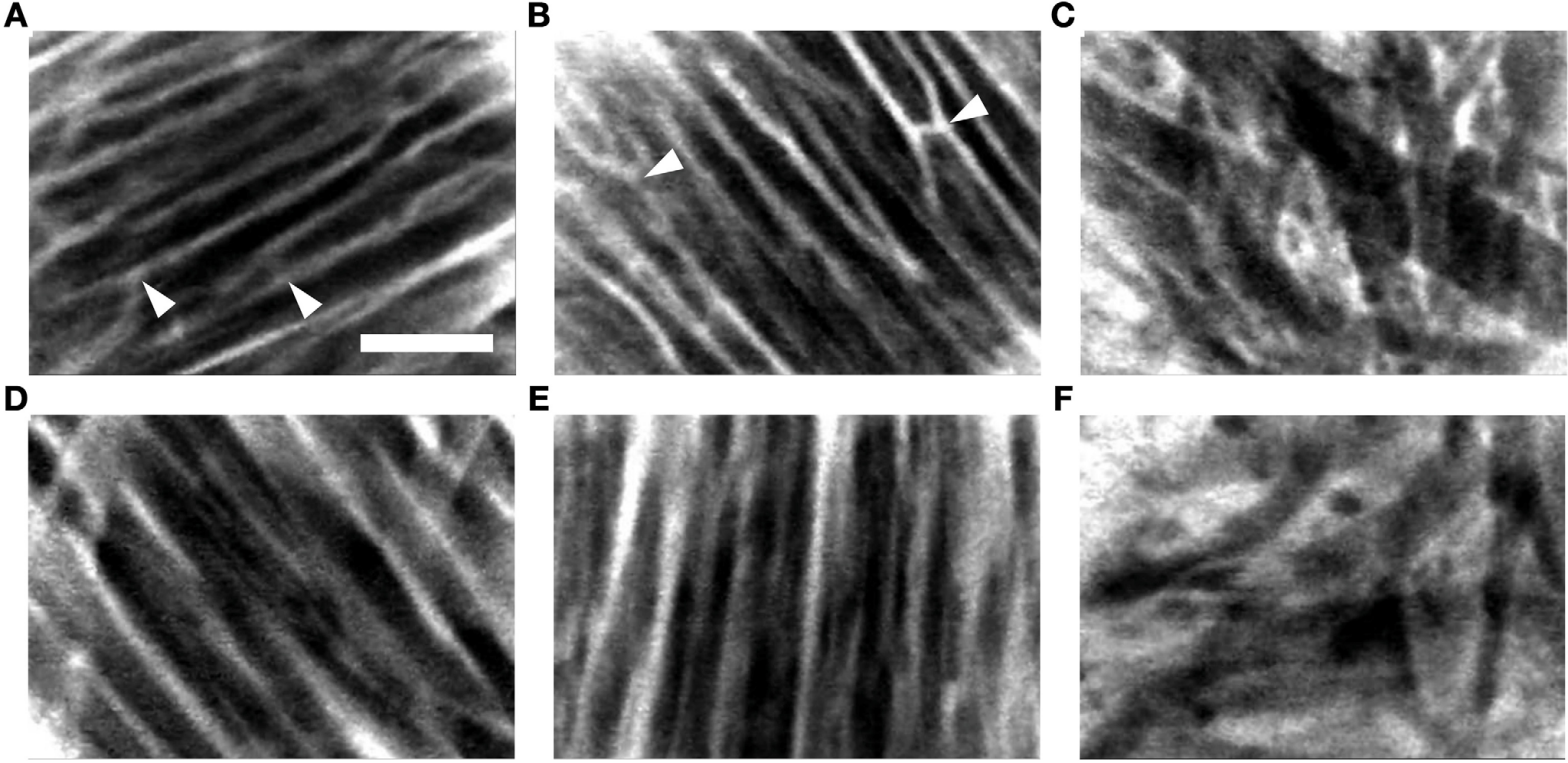
**Exemplary FCM image sequences acquired of the living arrested heart after administration of fluorescein sodium via (A–C) perfusion and (D–F) 1% foam-agarose dye carrier**. Images acquired following perfusion of fluorescein sodium in the **(A)** right ventricular and **(B)** right atrial subepicardial working myocardium displayed branching and transverse structures (arrowheads) characteristic of the microvascular bed. Structures of the microvascular bed were absent in images acquired of the **(D)** right ventricular and **(E)** right atrial subepicardial working myocardium following topical administration of fluorescein sodium via dye carrier. **(C,F)** Images acquired from the sinoatrial node region were also absent of microvascular structures respective of the dye delivery method. Scale: 50 μm.

In further experiments, we evaluated two methods of dye delivery in the living arrested heart. Figure 5 presents exemplary images acquired with the FCM from different regions in the heart after administration of fluorescein sodium via perfusion (Figures 5A–C) and 1% foam-agarose carrier (Figures 5D–F). Dark regions in these images suggest absence of fluorescence in intracellular spaces while the bright regions indicate presence of fluorescence within the extracellular space. A regular arrangement of intensities in the FCM images was visible in the right ventricular (Figures 5A,D) and right atrial (Figures 5B,E) regions. These images indicate working myocardium comprised of aligned myocytes. An irregular arrangement as seen in the sinoatrial node (Figures 5C,F) is a hallmark of reticular-arranged nodal cells (Huang et al., 2013). The microstructural arrangement in the working myocardium and in the sinoatrial node did not differ with respect to the method of dye delivery. However, images acquired using the perfusion method of dye delivery presented structural features of the microvascular bed in the working myocardium, including branching and transverse components (Figures 5A,B). These features were a frequent occurrence in the acquired movie sequences of the working myocardium following this delivery method (Videos 1, 2 in Supplementary Material). The microvascular components were not present in images acquired of the working myocardium using dye delivery via carrier (Figures 5D,E; Videos 4, 5 in Supplementary Material). Furthermore, microvascular features were not apparent in the images acquired from the sinoatrial node respective of the dye delivery approach (Figures 5C vs. 5F; Videos 3, 6 in Supplementary Material).

**Figure 6:**
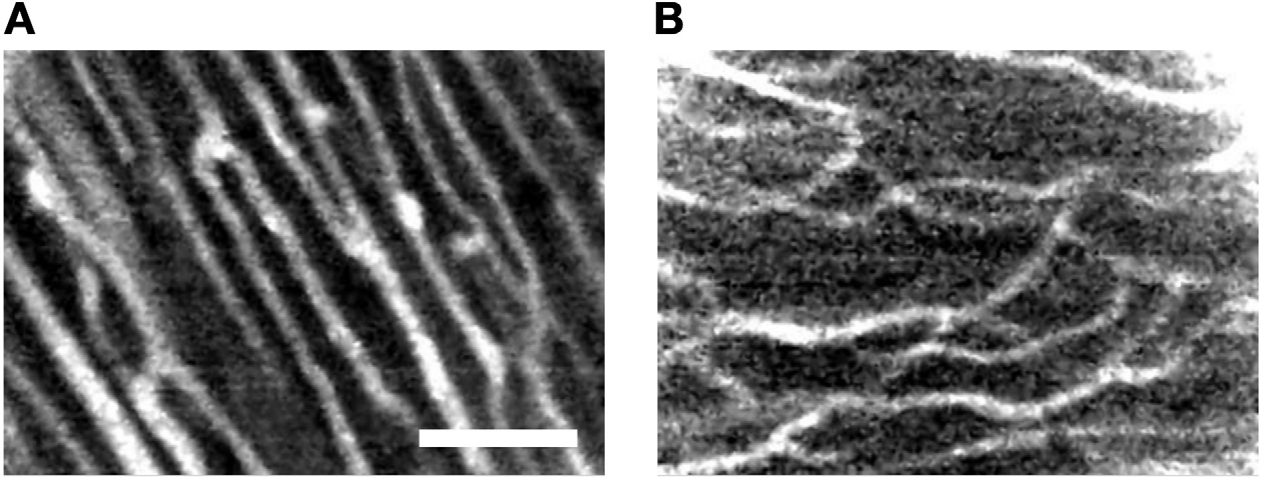
**Exemplary FCM images acquired of the living arrested heart after administration of high molecular weight dextran-conjugated fluorescein via perfusion**. The high molecular weight dextran-conjugate allowed for direct visualization of vasculature within the **(A)** right ventricular and **(B)** right atrial subepicardial myocardium. Microvasculature features of transverse and branching components are apparent. Scale: 50 μm.

We further explored imaging of the microvasculature in living heart experiments involving perfusion of 2 MDa dextran conjugated to fluorescein. Previous studies demonstrated that dextran conjugates greater than 40 KDa do not diffuse through either the endocardial endothelium or capillary endothelium in rat (Andries and Brutsaert, 1994) suggesting that this label provides reliable visualization of the microvasculature. Figure 6 presents exemplary FCM images of the microvasculature bed labeled with 2 MDa dextran-conjugate. The images present branching and transversal components similarly as observed in images of right ventricular (Figures 6A vs. 5A; Video 7 in Supplementary Material) and atrial working myocardium (Figures 6B vs. 5B; Video 8 in Supplementary Material) perfused with fluorescein sodium.

**Figure 7:**
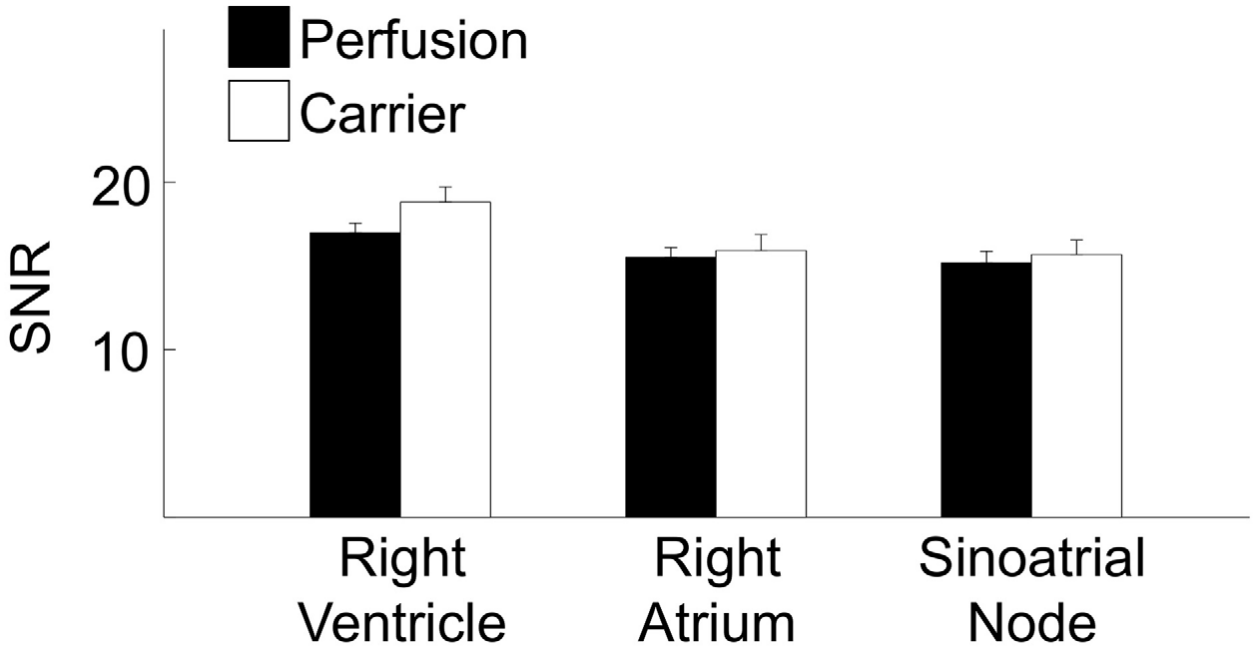
**Statistical characterization of FCM images from right ventricle, right atrium and sinoatrial node regions**. Differences in the SNR were not significant, respective of the dye delivery method (perfusion vs. carrier) or tissue region. (^*^P < 0.01, One-Way ANOVA with *post-hoc* Tukey–Kramer).

We quantitatively characterized the dye release of the evaluated dye delivery methods based on SNR (Table 2). Figure 7 presents statistical analysis of SNR from the previously acquired images in the living heart following dye carrier or perfusion dye delivery. The measured SNRs were in the range of 15–19. Differences in the SNR were not significant, respective of the dye delivery method or tissue region.

**Table 2:** **Number of Images and Animals Used for Signal-to-Noise Ratio Analysis**.

**Dye delivery**	**Images/animals**		
	**RV**	**RA**	**SN**
Perfusion	151/7	165/8	100/6
Carrier	74/6	53/5	55/6

*RV, indicates right ventricle; RA, right atrium; SN, sinoatrial node*.

## Discussion

FCM has emerged as a tool for basic biomedical research (Bharali et al., 2005; Vincent et al., 2006; Lewandowski et al., 2010) and clinical applications. Current clinical applications of FCM include imaging and diagnosis of the microstructure of tissues in gastrointestinal, respiratory, and urinary tracts. We recently proposed FCM for intraoperative tissue discrimination during cardiac interventions (Huang et al., 2013), in particular, for pediatric open-heart surgery. In these cardiac interventions and many other clinical applications FCM imaging relies on intravenous injection of high-dose fluorescein sodium as a fluorescent marker. While concerns for patient safety associated with this approach for delivery of fluorescent dye are minor, we believe that alternative reliable methods for dye delivery can be introduced.

Here we developed and evaluated local dye delivery as an approach for FCM imaging in the living heart. We demonstrated that a foam-agarose dye carrier comprised of porous foam and agarose hydrogel reliably delivers fluorescent dye in sufficient concentration for FCM. Our evaluations showed that foam-agarose dye carriers exhibit a prolonged dye release compared to foam-only carriers. A probable cause is the dense cross-linked network of agar strands within the foam pores, which impede diffusion of dye inside and out of the carrier.

Diffusion of molecules, solutes, and particles in agarose gels has been extensively studied (Johnson et al., 1996; Pluen et al., 1999; Fatin-Rouge et al., 2004). Based on those studies, we expected differences of diffusion and dye release due to differences in the Stokes radius of fluorophores. We also expected differences of dye release due to differences in the agarose concentration. However, dye release was in general not affected by agarose concentration between 1 and 5% and the type of dye. We found only a single combination of agarose concentration and dye exhibiting prolonged dye release (Figure 4C). While we cannot explain this prolonged release, our finding suggests that dye release properties can be optimized for specific dyes.

Cyclic line tests on synthetic tissue analogs demonstrated that foam-agarose dye carriers allowed for reliable imaging of at least 5–9 lines and in the case of the 3% carrier loaded with fluorescein sodium up to 18 lines. Based on the fact that scan times for each line of a cyclic line test was 50 s, we estimate that the foam-agarose carriers allows for continuous dye release of up to 15 min for the 3% carrier loaded with fluorescein sodium and between 4 and 8 min for the other foam-agarose carriers. We expect that dye release over these durations will be sufficient for many clinical applications, when one considers practical limitations of the length of surgical interventions. For applications of FCM in cardiac interventions, we expect that the imaging window can be up to minutes in order to reduce burden on patients where time on cardiopulmonary bypass is of crucial importance.

Becker et al. investigated the optimal time window for FCM imaging of the upper gastrointestinal tract in a porcine model following intravenous injection of fluorescein sodium (Becker et al., 2008). The study revealed that contrast and image quality was high between 1 and 8 min after injection of fluorescein sodium. The results of our cyclic line tests suggest that local dye delivery based on foam-agarose carriers produces imaging windows of similar duration. An advantage of the dye carrier over systemic injection is that the FCM imaging can start instantaneously without delays. In contrast, the transit times of fluorescein sodium from initial injection to detectable fluorescence was reported to be 10–20 s for retinal fluorescein angiography (Duane et al., 1991; Eskridge et al., 1991) and 30 s for gastrointestinal endoscopic imaging (Becker et al., 2008).

Our study revealed that the SNR of images acquired from the living heart following both local and perfusion delivery of fluorescent dyes are not different. In addition, the observed SNRs were comparable to the SNRs of 10–20 reported in studies involving FCM imaging of the human gastrointestinal tract with intravenous fluorescein sodium (Shahid et al., 2011). This further suggests that local dye delivery approaches for FCM produce images of similar quality to that of systemic delivery. However, an important difference between local and systemic dye delivery was related to the imaged tissue microstructure. We observed that the perfusion method of dye delivery presented structural features characteristic of microvasculature in images acquired from working myocardium (Brown, 1965; Chilingaryan et al., 2014). These features were not observed in images acquired following local dye delivery. Also, these structures were not visible within the sinoatrial node region for both methods of dye delivery. We hypothesize that the differences in the images of tissue microstructure are explained by differences of dye transport to the imaged region. In the perfusion method of delivery dye is transported through coronary vessels and microcirculation of the Langendorff-perfused heart. Therefore, we expect a diffusion gradient with the highest and lowest concentrations of dye within the microvasculature and intracellular space, respectively. In addition, fluorescein predominantly stays in the vessels as 70–80% of it is bound to plasma albumin. Only a small portion of fluorescein, i.e., unbound fluorescein, eventually leaks into the tissue interstitium and allows for visualization of cellular morphology (Becker et al., 2008). Our imaging studies using a high molecular weight dextran-conjugated fluorophore as perfusate supported this hypothesis. The high molecular weight dextran-conjugate selectively labeled the microcirculation identified by branching and transversal components within the tissue (Figure 6). Thus, images of working myocardium following systemic application of fluorescein sodium appear to be a superposition of fluorescent signal from the microcirculation and extravascular space. In contrast to the perfusion method of dye delivery, local dye delivery in the heart relies on diffusion of dye through the epicardium or endocardium into the sub-epicardial or sub-endocardial myocardium. The highest concentration of dye in the tissue is within the interstitial space. Dye that diffuses into the vasculature is quickly removed from the labeled region, which leads to absence of vascular signals. Our findings suggest that the type of microstructural features to be visualized with FCM should determine the approach for dye delivery. For instance, discrimination of cardiac tissue types during open-heart surgery requires visualization of the microstructural arrangement of cellular features. In this case, the local dye delivery approach appears more appropriate, because images are less affected by the distribution of microvasculature. If the application is characterization of microvascular features then systemic application of the appropriate fluorescent dye is preferred. Our findings also suggest that the degree of vascularization within the imaged region may limit systemic delivery approaches for FCM. For instance, scarred regions in infarcted or fibrotic hearts are known to be poorly perfused (Kim et al., 1996; Saeed et al., 2006). Thus, a local dye application may be more suitable for visualization of myocardial scars.

Our studies revealed major differences in the dose of fluorescein sodium required for reliable FCM imaging in the living heart using the two dye delivery approaches. The typical dose for intravenous administration of fluorescein sodium is either 200 or 500 mg at a concentration of 100 mg/mL. In recent years, it has been reported that the 500 mg dose produces superior images to that of the 200 mg dose in both retinal fluorescein angiography (Moosbrugger and Sheidow, 2008) and gastrointestinal endoscopic imaging (Shahid et al., 2011) studies. Based on weight measurements we estimate that a fully loaded dye carrier can hold approximately 4.1 μg of fluorescein sodium. In contrast, our model of systemic delivery required perfusion of the heart with approximately 30 μg/min. The comparison indicates that local dye delivery requires significantly less dye than systemic delivery. We suggest that local dye delivery is in particular suitable for clinical applications such as pediatric open-heart surgery where microdosing will mitigate potential safety concerns.

In a previous study, we introduced a dye carrier based on hydrogel indicating feasibly of local dye delivery (Lasher et al., 2009; Sachse et al., 2011). This dye carrier comprised a hollow hydrogel cylinder serving as dye reservoir and a thin layer of transparent hydrogel directly in front of the microprobe allowing for diffusion of dye into the tissue. The dye carrier necessitated an imaging microprobe with a large focal depth to accommodate for the addition of hydrogel in front of the lens. Also, this carrier required precise fabrication of the hydrogel layer at micro-meter scale. Furthermore, the precise attachment of the hydrogel at the microprobe tip and the overall fragility of hydrogel complicated handling. In comparison, fabricating the presented foam-agarose dye carrier is simple and does not require precision at micro-meter scale. The foam-agarose dye carrier was designed for lateral attachment to the imaging probe and does not occupy space in front of the microprobe. The combination of foam with hydrogel also improved the mechanical stability of the dye carrier.

Many limitations of FCM are related to technological and physical barriers of conventional confocal microscopy. An overview of limitations of confocal microscopy can be found, for instance, in Bolte and Cordelières, (2006). One limitation of confocal microscopy is that only regions close to the surface of the tissue can be imaged. This is an inherent problem of optical microscopy related to absorption and scatter of light by biological tissue. Using current FCM systems we expect that imaging of tissue will be possible to a depth of 100 μm, which appears sufficient to gain important research and diagnostic information. The spatial resolution of current FCM systems is smaller than the resolution of high-end conventional confocal microscopes, having for instance, oil immersion lenses with a high numerical aperture. The low resolution of current FCM systems makes it difficult to identify cell ends or the subcellular micro-structure mentioned above. However, the presented image data (Figure 5) suggest that important features of cardiac tissue can be identified, for instance, related to the regularity of the distribution of the labeled extracellular and vascular space.

Certain limitations are associated with using Langendorff perfusion of fluorescent dye in Tyrode's solution as a model of systemic dye delivery. Intravenous injection applies a bolus for fluorescent dye into the systemic circulation. The dose that each organ or tissue receives and how long the dye remains in the different regions varies. Our simple model of systemic dye delivery was based on observed fluorescent intensities. We chose a concentration and flow rate necessary to reconstruct intensities from previous studies (Huang et al., 2013). An option for future studies is to perform those in the anesthetized animal after thoracotomy. While we expect that higher dye concentrations for systemic delivery will be required due the binding of fluorescein to plasma albumin (Becker et al., 2008), we do not anticipate that changes in the concentration of dye in the foam-agarose carrier are necessary for imaging of cardiac tissues.

In our study we were not able to identify vasculature within the sinoatrial node region, which is in contrast to previous reports that the sinoatrial node has a rich intramural network of anastomosing blood vessels and that the density of the microvasculature in the node is greater than in adjacent atrial tissue (Ovcina and Cemerlic, 1997). A possible explanation is the focal depth of the applied probe (approximately 26 μm). It is possible that vasculature in the sinoatrial node resides in the depth of the atrial wall that is not accessible with this imaging probe. It is also possible that visualization of the dense and complex network of the microvasculature is difficult in context of the dense and irregular cellular arrangement found within this nodal region.

In conclusion, our studies demonstrate that the introduced local dye delivery approach for FCM imaging constitutes an important alternative to commonly applied systemic approaches of dye delivery. We suggest that the approach for local dye delivery will facilitate clinical translation, for instance, for FCM imaging during pediatric heart surgery. Furthermore, we believe that the foam-agarose dye carrier and methodology for evaluation of dye release have significant applications in local delivery of other fluorophores and development of further types of dye carriers.

## Author contributions

Substantial contributions to the conception or design of the work; or the acquisition, analysis, or interpretation of data for the work: Robert W. Hitchcock, Chao Huang, Aditya K. Kaza, Frank B. Sachse. Drafting the work or revising it critically for important intellectual content: Robert W. Hitchcock, Chao Huang, Aditya K. Kaza, Frank B. Sachse. Final approval: Robert W. Hitchcock, Chao Huang, Aditya K. Kaza, Frank B. Sachse. Agreement to be accountable for all aspects of the work in ensuring that questions related to the accuracy or integrity of any part of the work are appropriately investigated and resolved: Robert W. Hitchcock, Chao Huang, Aditya K. Kaza, Frank B. Sachse.

### Conflict of interest statement

A patent related to FCM imaging of tissues and dye application has been issued (Dye application for confocal imaging of cellular microstructure, US2013/057419, University of Utah Research Foundation). A patent application related to fluorescence imaging of tissue is pending (Devices and systems for fluorescence imaging of tissue, US2013/022247, University of Utah Research Foundation). The authors declare that the research was conducted in the absence of any commercial or financial relationships that could be construed as a potential conflict of interest.
