# Supplementary material for: Local delivery of fluorescent dye for fiber-optics confocal microscopy of the living heart
Source: Front Physiol. 2014 Sep 25;5:367. doi: 10.3389/fphys.2014.00367 (PMC4174735; doi:10.3389/fphys.2014.00367)
Supplement: Supplementary file 9 [file DataSheet1.PDF]

Movie Legends

Movie I. Exemplary FCM image sequence acquired from the right ventricular working myocardium following Langendorff perfusion of an isolated rat heart with fluorescein sodium. Fluorescent features characteristic of microvasculature such as branching and transverse structures appear frequently throughout the image sequence. Scale: 20 μm.

Movie II. Exemplary FCM image sequence from the right atrial working myocardium acquired following perfusion dye delivery. Similar to Movie I, components of the microvasculature are clearly present. Scale: 20 μm.

Movie III. Exemplary FCM image sequence from the sinoatrial node following dye perfusion reveals reticular microstructural arrangements indicative of this region. Scale: 20 μm.

Movie IV. Exemplary FCM image sequence acquired from the right ventricular working myocardium following dye delivery via foam-agarose carrier with 1% agarose concentration loaded with fluorescein sodium. Unlike perfusion dye delivery, microvasculature structures were not apparent using this local dye delivery method. Scale: 20 μm.

Movie V. Exemplary FCM image sequence from the right atrial working myocardium. The same imaging and dye delivery protocol for Movie IV was used. Scale: 20 μm.

Movie VI. Exemplary FCM image sequence from the sinoatrial node. Reticular arranged structures are visible. The same imaging and dye delivery protocol for Movie IV was used. Scale: 20 μm.

Movie VII. Exemplary FCM image sequence from the right ventricular working myocardium following perfusion of 2 MDa dextran-conjugated fluorescein. Fluorescent signal is present within structures characteristic of microvasculature. Scale: 20 μm.

Movie VIII. Exemplary FCM image sequence from the right atrial working myocardium. The same imaging and dye delivery protocol for Movie VII was used. Scale: 20 μm.
